# Tri-Valvular Endocarditis in a Previously Normal Heart Misdiagnosed as Recurrent Valley Fever

**DOI:** 10.7759/cureus.8678

**Published:** 2020-06-17

**Authors:** Anup Solsi, Andrew Talon, Pallavi Bellamkonda

**Affiliations:** 1 Internal Medicine, Creighton University Arizona Health Education Alliance/Valleywise Health Medical Center, Phoenix, USA; 2 Cardiovascular Disease, Creighton University School of Medicine - St. Joseph's Hospital and Medical Center, Phoenix , USA

**Keywords:** septic emboli, infective endocarditis, multiple valves, coccidioidomycosis, transthoracic echocardiogram, streptococcus viridans, modified dukes criteria

## Abstract

Infective endocarditis (IE) is classified as an infection of any cardiac valve or endocardial surface. This condition is associated with high morbidity and mortality; hence, early diagnosis and rapid intervention are extremely vital. Although IE is frequently found to infect only one heart valve, rare instances have shown multi-valvular involvement. Many conditions can present similar to IE, often delaying the diagnosis of IE. Coccidioidomycosis (or Valley Fever), a fungal infection endemic in the Southwestern United States, can present with features analogous to those of IE. We present the case of a middle-aged male with no underlying structural heart disease found to have tri-valvular IE after being misdiagnosed with recurrent Valley Fever.

## Introduction

Infective endocarditis (IE) is an acute or subacute infection of the endocardium of the heart with predilection for heart valve involvement. Overall incidence is approximately 3-10 per 100,000 persons, with recent global mortality rates ranging from 4% to 48% [1-2]. Thus, early diagnosis and prompt intervention are extremely vital. Predisposing risk factors of IE include structural heart disease, presence of prosthetic valves or cardiac devices such as pacemakers or defibrillators, IV drug use, immunosuppression, or recent invasive procedures [3]. Gram-positive cocci such as staphylococcus, streptococcus, and enterococcus species account for 80%-90% of IE cases, with *Staphylococcus aureus* and coagulase-negative staphylococci comprising 25% and 22% of cases, respectively [1, 4]. IE is frequently associated with an assortment of immunologic (Roth spots, glomerulonephritis, Osler’s nodes) and vascular phenomena (septic emboli, mycotic aneurysms, pulmonary infarcts, Janeway lesions), which may obscure the diagnosis of IE. IE can masquerade as a variety of diagnoses, necessitating a broad diagnostic workup and evaluation. Complications from IE can occur frequently, most commonly cardiac in origin, usually leading to overt heart failure from infection-induced valvular damage and subsequent valvular insufficiency [5].

The majority of IE cases typically involve only one valve, with two valve involvement being less frequent and triple or quadruple valve involvement reported to be extremely rare [6]. Interestingly, mortality rates seem to be similar between single and multiple valve IE, however, multivalvular involvement has an increased rate of congestive heart failure (CHF) complications requiring surgical management [7-8].

Here, we present the unique case of a previously healthy male with no underlying structural heart disease found to have tri-valvular endocarditis after being initially misdiagnosed with recurrent Valley Fever.

## Case presentation

A 57-year-old male from Arizona with a medical history significant for previously treated coccidioidomycosis, presented to the ED with progressively worsening shortness of breath over the prior four months, with recent fevers, chills, night sweats, and back pain. He denied the use of injection drugs, such as heroin or methamphetamine. On examination, the patient was afebrile, but tachycardic, tachypneic, and hypertensive. Cardiovascular examination revealed jugular venous distension and a grade 3/6 holosystolic murmur at the left sternal border. He showed no peripheral stigmata of IE, no Osler nodes, Janeway lesions, or splinter hemorrhages. Bibasilar crackles were heard on auscultation of the lungs. He was noted to have tenderness over his lower thoracic vertebrae with limited range of motion. Laboratory investigations revealed leukocytosis and microcytic anemia. Erythrocyte sedimentation was 68 mm/h, Troponin I was elevated at 0.52 ng/mL, and brain natriuretic peptide (BNP) was 997 pg/mL. Serum electrolytes and renal function were within normal limits. Apart from a positive rheumatoid factor, autoantibody screening was negative. Electrocardiogram (ECG) demonstrated a normal sinus rhythm with left axis deviation.

**Figure 1:**
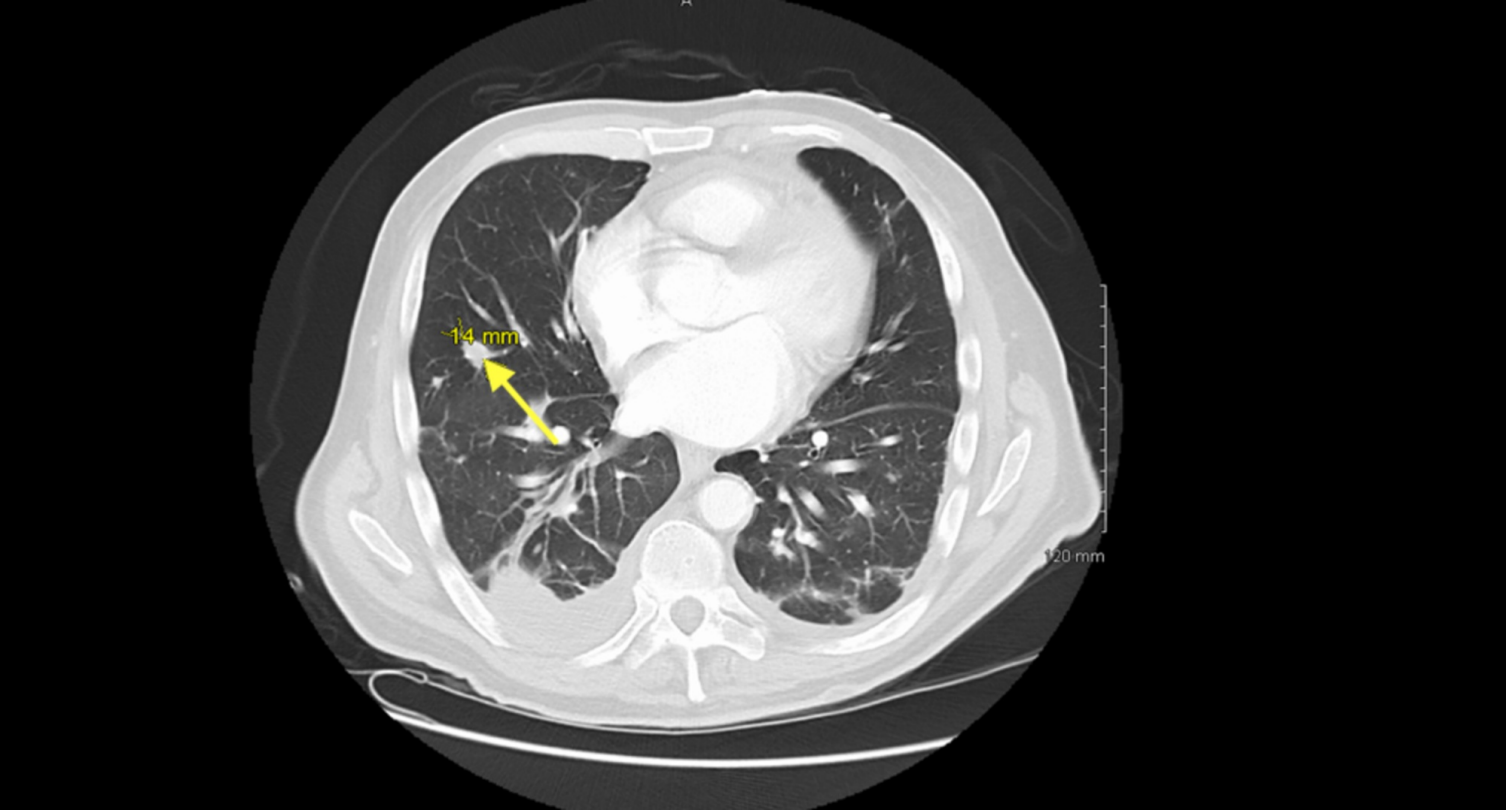
CTA of chest showing multiple pulmonary defects and multiple pulmonary nodules (arrow shows 14 mm nodule) scattered throughout both lobes. CTA, computed tomography angiography

Due to suspicion of recurrent coccidioidomycosis, the patient was initially treated with fluconazole. Serologies returned negative, with complement fixation titer <1:2 as well as nonreactive IgG and IgM immunodiffusion. For further evaluation, a computed tomography angiography (CTA) chest was obtained, which exhibited a small right-sided pleural effusion as well as multiple pulmonary nodules and cavitary lesions consistent with septic emboli (Figure 1).

**Figure 2:**
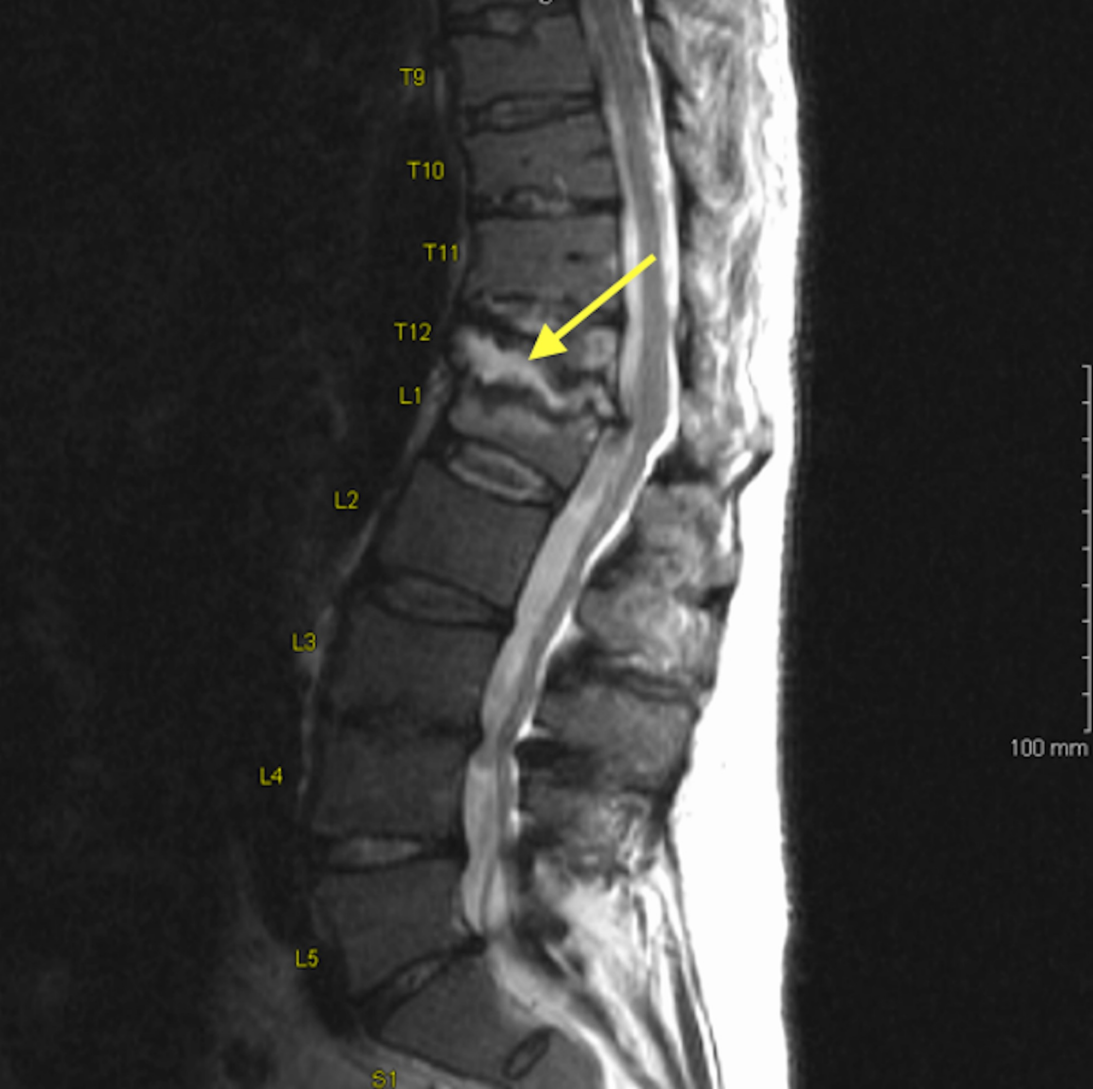
MRI of lumbar spine showing degenerative changes at T12/L1 level consistent with spondylodiscitis with vertebral osteomyelitis (arrow).

Additional imaging in the form of a MRI of the spine was done for further evaluation of the back pain, which showed spondylodiscitis with vertebral osteomyelitis at the T12/L1 level (Figure 2). A bone biopsy was not performed.

**Figure 3:**
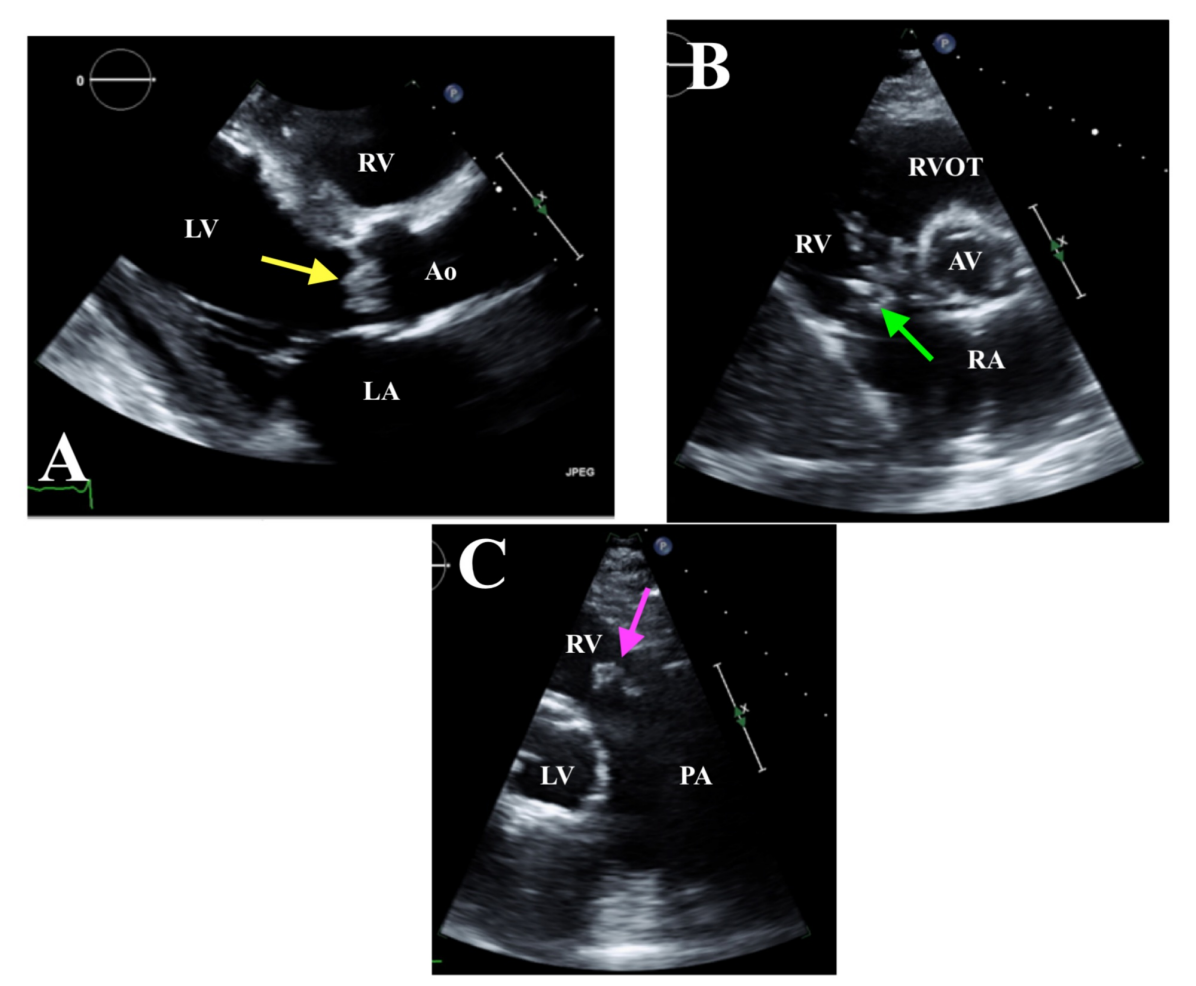
TTE. A: Parasternal long axis view with AV vegetation measuring 0.9 cm x 0.8 cm (yellow arrow). B: Parasternal short axis view showing tricuspid valve vegetation measuring 1.4 cm (green arrow). C: RVOT view with pulmonic valve vegetation measuring 1.4 cm x 1.1 cm (pink arrow). TTE, transthoracic echocardiogram; LA, left atrium; LV, left ventricle; Ao, aorta; AV, aortic valve; RV, right ventricle; PA, pulmonary artery; RVOT, right ventricular outflow tract

Given the fevers and CTA imaging findings suggestive of septic emboli, a transthoracic echocardiogram (TTE) was performed. TTE revealed biventricular dysfunction with an estimated ejection fraction of 25%-30%; multiple vegetations were seen on the aortic, tricuspid, and pulmonic valves measured at 0.9 cm x 0.8 cm, 1.4 cm, and 1.4 cm x 1.1 cm, respectively (Figure 3A-C).

As there was clear evidence of multiple valvular vegetations, transesophageal echocardiogram (TEE) was not performed. Empiric IV antibiotic therapy with vancomycin and piperacillin/tazobactam was initiated. Fluconazole was discontinued. Blood cultures returned positive for *Streptococcus mutans*, sensitive to ceftriaxone. Antibiotic regimen was modified accordingly, and the patient was started on a six-week course of IV ceftriaxone and continued on vancomycin due to concerns for methicillin-resistant *Staphylococcus aureus* (MRSA). Ensuing panoramic radiography revealed extensive dental disease. After a prolonged hospital stay, the patient was subsequently transferred to a tertiary care center for surgical evaluation. As the patient was lost to follow up, it is unclear if surgical valve repair/replacement was performed or if he completed the course of antibiotics.

## Discussion

The use of the Modified Duke Criteria is often employed to assist in the diagnosis of IE based on clinical features, microbiological investigation and echocardiographic findings, with a definitive diagnosis requiring two major or one major and three minor or five minor criteria [9]. Our patient met the Modified Duke Criteria diagnosis for IE by satisfying two major criteria -- a TTE with valvular vegetations suggestive of IE and blood cultures positive for *S. mutans*, a viridans group streptococci, considered a typical microorganism for IE. Viridans streptococcal species are among the most common causative organisms of IE, with some estimates suggesting around 50% of all cases [10]. Many of the viridans streptococcal bacteria are part of normal microbial flora and are most prevalent in the oral cavity, the likely source in our patient with dental disease. Typically, viridans streptococci is found to cause left-sided sided IE [11]. Our patient was noted to have tri-valvular IE, with one of the valves being the tricuspid valve. Infection of this valve is commonly seen in patients using IV drugs, which our patient denied. It remains unclear why viridians group streptococci have an affinity for the aortic and mitral valve.

Results of 2D imaging with TTE is a major contributor to making a diagnosis of IE. In suspected native valve IE, TTE has a sensitivity of 75% and a specificity of 90% for detecting vegetations [12]. If the TTE is equivocal or negative for vegetations, but suspicion remains high for IE, the more invasive TEE should be done, as it has a sensitivity of over 90% for identifying valvular vegetations [1]. 

Multivalvular endocarditis (MVE) has rarely been reported in medical literature, with little data regarding its characteristics, best management approach, and even prognosis. The incidence of MVE varies from 12.4 % to 31% [6, 13]. A prior study investigating 77 patients with MVE found viridans streptococci to be the most frequent cause of MVE with an overall mortality of MVE of 21%, comparable to the ~18% mortality seen in single-valve IE cases [6]. Initial management of MVE is similar to that of single valve IE, with the use of IV antibiotics for a prolonged four to six week duration. However, 40%-50% of patients with IE require surgical intervention, with congestive heart failure (CHF) from valvular obstruction and regurgitation being the most common indication [1,14]. Furthermore, CHF has been shown to be the most frequent complication of patients with MVE, which suggests surgical treatment is crucial in managing cases of MVE [13]. The European Society of Cardiology and American College of Cardiology/American Heart Association guidelines currently recommend surgical management in the presence of heart failure, severe valvular regurgitation, perivalvular abscess or fistula, embolic events, presence of large vegetations, or signs of uncontrolled infection [15]. In a landmark trial from 2018, early surgery (within 48 h) compared to medical management resulted in a statistically significant reduction in in-hospital death and embolic events within six weeks in the early surgery group [16]. Unfortunately, no studies have documented the survival implications an early surgical approach has on patients with MVE. Contraindications to surgical treatment include large tricuspid vegetations, severe tricuspid regurgitation with poor response to therapy, or prolonged bacteremia [17]. Further research into MVE is needed in order to assist clinicians in managing this rare condition.

The diagnostic challenge in our case was our patient had a history of coccidioidomycosis, a fungus causing infection endemic in the Southwestern United States [18]. Infection occurs when spores are inhaled from the soil, causing most patients to present with pulmonary symptoms, with 5%-10% of cases resulting in pulmonary sequelae, which may include nodules or peripheral thin-walled cavities demonstrated on imaging [18]. The pulmonary symptoms coupled with other nonspecific symptoms such as fever and fatigue can be misleading, as IE and coccidioidomycosis share symptomology. As infection disseminates, bacteremia and systemic complications can occur, making it nearly indistinguishable from symptoms of IE. The embolic manifestations of IE on this CT scan showing multiple septic emboli and pulmonary nodules are similar to the appearance of coccidioidomycosis on imaging, which consequently led to a delay in diagnosis and the initiation of the correct antimicrobial therapy. Although coccidioidomycosis recurrence is relatively uncommon, the radiographic findings here were consistent with a primary pulmonary infection. In an endemic area such as Arizona, clinical suspicion for coccidioidomycosis should always remain high; particularly in a patient presenting with shortness of breath and constitutional symptoms. However, negative serologies should warrant alternative differentials, such as IE. 

## Conclusions

Even though diagnostic criteria have been developed, diagnosis of IE can often be challenging, as many systemic illnesses can present with similar symptoms. IE should be especially considered in coccidioidomycosis endemic areas and must be investigated for once a diagnosis of coccidioidomycosis is excluded. If there is even the slightest concern for IE, we recommend prompt evaluation with TTE and possibly TEE to identify valvular lesions, keeping in mind multiple valves may be involved. If symptoms of overt heart failure or complications from septic embolic are present, immediate surgical consultation may prove to be a life-sustaining measure. 
